# NK cells blur the frontier between innate and acquired immunity

**DOI:** 10.3389/fimmu.2012.00400

**Published:** 2013-01-02

**Authors:** Giuseppe Terrazzano, Ennio Carbone

**Affiliations:** ^1^Department of Science, University of BasilicataPotenza, Italy; ^2^Department of Cellular and Molecular Biology and Pathology, University of Naples Federico IINapoli, Italy; ^3^Department of Experimental and Clinic Medicine, University of Magna Graecia of CatanzaroCatanzaro, Italy; ^4^Department of Microbiology Cell and Tumorbiology, Karolinska InstitutetStockholm, Sweden

Since their original description in 1970s, huge steps forward have been made on the knowledge of the biology of Natural Killer lymphocytes (NK). Several evidences indicate that NK could be considered as a keystone in the immune system regulation, indeed they bring together the innate and adaptive immunity, branches of the immune response.

Hitherto, adaptive immune response has been classified according to three main paradigms: the recombination of specific gene for the receptor repertoire (i.e., RAG1/2 gene expression for B cell receptor (BCR) and T cell receptor (TCR), the acquirement of specificity and immune memory due to the interaction with antigen processing cells (APC) in secondary lymphoid organs, and the timing of action; moreover, while innate immunity receptors do not require gene rearrangement, adaptive immunity is quickly acting and have no immune memory.

In this context, the more orthodox point of view separates, according to their functions, the innate from the adaptive immune responses. Nevertheless, several data's suggest a fine integration of the two arms of immunity. This integration is promoted by the interaction and involvement of many cells “acting in concert” to achieve the protection of “self” biological-components. In this regard, it is worth noting that NK cells can even work as “adaptive effectors” once activated by the IFN-γ produced by T cells or when the IgG bounds to CD16 which eventually elicits an antibody dependent cell cytotoxicity, (ADCC) response (Lanier, 2005; Caligiuri, 2008; Vivier et al., 2008).

The common opinion is that innate response discriminate the self from non-self recognizing microbial products (such as bacterial or fungal LPS, etc.). These components recognized by germline encoded receptors as “non-self,” are associated with pathogens. Moreover, the adaptive immunity surveils and preserves the integrity of the self, recognizing a wide range of alterations i.e., non-self allogeneic transplanted tissues, self-altered tissues, which might be altered either by virus or oncogene-derived peptides. The adaptive cells recognize MHC molecules by RAG1/2-dependent receptors, TCR.

While the innate immunity plays a crucial role in the first 48–72 h of the pathogenetic event, adaptive immune surveillance requests longer time (weeks or months) and the related immunological memory could have no expiration throughout life. This dichotomous view can be unified whether one considers that immunity is a complex of integrated responses, finalistically linked for the elimination of pathological intruders and the preservation/restoration of the organism homeostasis. This hypothesis may lead to many philosophical implications, but also suggests a different point of view in order to understand the biology of those cells that are necessary for the integration between the innate and acquired responses. In such scenario, the recent acquisition of knowledge about the functions of NK spread new light on the effects of these cells in the regulation of the whole immune response and their role in adaptive immune response (Paust et al., 2010).

The aim of this opinion is to focus the reader's attention toward the “holistic” regulatory effects of NK cell in immune response: this feature of the NK cell biology field is till now neglected, but it is highly promising for a fertile research and translational applications.

## The NK receptors multiciplity

NK cells receptor repertoire shares features with macrophages as the recognition of PAMPs, Fc, and MIP1-alpha thanks to TLR, CD16, and CCR5 (Lanier, 2005; Caligiuri, 2008; Vivier et al., 2008). Surprisingly, in the last decades NK cellular programs were found to be regulated by MHC class I molecules in a reciprocal way of the one used by CD8 + CD3 + T cells via TCR recognition (Kärre et al., 1986).

Intensive research in the 1990s clarified the molecular basis of their regulation of cytotoxic activities (Caligiuri, 2008), making them a promising new tool for biological therapy in cancer (Ljunggren and Malmberg, 2007; Velardi, 2012).

The presence of a multiplicity of receptors, such as TLR, ancient lectin-like receptors (CD94), (Lanier, 2005; Caligiuri, 2008; Vivier et al., 2008), KIRs (Vivier and Anfossi, 2004; Lanier, 2005; Parham, 2005; Caligiuri, 2008; Vivier et al., 2008), CD40, CD80, CD86 receptors (Carbone et al., 1997, 1999; Martín-Fontecha et al., 1999; Terrazzano et al., 2002), stress inducible molecules receptors (Gleimer and Parham, 2003) gave to NK cells a high plasticity in their interaction with several other immune system cell types. These features already predict their functional plasticity, making them hard to classify as innate or adoptive immunity cell type.

## Multiple NK cells mediated interactions within immunity

Since their discovery (Kiessling et al., 1975), NK cells have been classified as innate cytotoxic effectors recognizing tumors and viral-infected cell targets using classical cell cytotoxicity assays. Hitherto, these kinds of experiments have been the leitmotiv of the NK cells-related research. In this regard, only in the recent past it has been suggested a role for NK activity in the regulation of the balance adaptive immunity (Vivier and Anfossi, 2004; Lanier, 2005; Parham, 2005; Caligiuri, 2008; Vivier et al., 2008). In spite of these achievements, NK cells are holding peculiar immunological features which still are unknown. On the other hand, their role in the adoptive immune response regulation was already described in the 1980s (Kuwano et al., 1986; Tovar et al., 1986). Moreover, the interaction between adaptive tumoral response and NK cells is evident since IgG2a is the key adoptive effector molecule that triggers NK cells ADCC by CD16 engagement. The relevance of this adaptive NK activation pathway is witnessed by the dominant role of CD16 NK receptor that does not require co-stimulatory signal by the other activating receptors (Bryceson et al., 2009). NK cells have been reported to interact with B cells (Procopio et al., 1985; Wyatt and Dawson, 1991) DC (Carbone et al., 1999, 2000; Ferlazzo et al., 2002; Moretta, 2002) with T cells (Ardolino et al., 2011), and with granulocytes (Jaeger et al., 2012).

## The NK cells memory revealed in pathology

NK memory has been recently described after viral infection by CMV in human and mouse, HIV and hantavirus in human (Salazar-Mather et al., 1998; Björkström et al., 2011; Eller et al., 2011; Lopez-Vergès et al., 2011). So far the life span of this memory NK subset has not been investigated in detail. Nevertheless, for HIV and CMV infected patients it is conceivable retain that the NK cells memory can last for years, far over the classical innate immunity time window.

## The NK cells in secondary lymphoid tissues

NK cells are in the lymph nodes, and human CD56 bright NK cells have been found to be activated by CD8+T-derived IFN-γ; eventually these T-cell activated NK prime DC maturations (Caligiuri, 2008). Murine NK cells are quickly recruited in lymph nodes after T cells immunization and they contribute to Th1 immune polarization (Martín-Fontecha et al., 2004). Analysis by *in vivo* imaging evidenced that NK cell, during leishmania major infection, after FN-γ production, could influence both the DC and T cells adaptive activity (Bajénoff et al., 2006). A recently described NK subset is able to produce IL-22 and IL-17 and seems to be involved in lymphoid tissue organogenesis, which was thus named “lymphoid tissue inducer cells” (LTi) (Cupedo et al., 2009). This original NK cell appears different from to classical NK (Crellin et al., 2010) and it can develop in NK22 cell subset described to play a major role in regulating T and B mucosal immunity (Cella et al., 2010). Therefore, NK cells are present in secondary organs where they play a regulatory role for the immune response.

## The NK cells tolerance

Despite the involvement of NK cells in self-tolerance has been suggested in autoimmunity disease, their role is still controversial and not clearly understood (Lunemann et al., 2009). NK cells appear to either protect or increase autoimmune disease. In animal models, they protect against autoimmunity in early phase of inflammation, but they exacerbate autoimmune diseases when the chronic inflammation is fully consolidated (Bielekova et al., 2006). In particular, depletion of NK cell increases autoimmune disease, whereas their adoptive transfer reduces the severity of the disease (Ogasawara et al., 2003, 2004; Li et al., 2005). In humans, predisposition to several autoimmune diseases (Martin et al., 2002; Luszczek et al., 2004; Momot et al., 2004; Suzuki et al., 2004) has also been associated to the expression of certain KIRs and HLA genes. In myelodysplastic syndromes, it has been suggested a role for NK in the selection/expansion of dysplastic clones (Ruggiero et al., 2009; Terrazzano et al., 2012). Thus, in the autoimmune diseases NK cells play a role mainly as regulatory cells for aberrant and detrimental immune response.

## Should new experimental approaches *in vitro* and *in vivo* be considered?

In reason of what has been discussed, it is conceivable to propose new approaches for the understanding of NK biology. The above reported considerations could be empirically tested using experimental design where NK cells are investigated in concert with other immune cells.

### In vitro

So far, the read out for NK activities has been the study of cell-mediated cytotoxicity, degranulation assays, or IFN-γ production. This approach gives sharp informations on the possible NK cell programs, but does not explore universally the relevancy of NK cells in immune response. In contrast, a “three cells” experiment (i.e., NK/DC/T or B cells) could help us finding out the ultimate effects of NK cells regulation on immunity during infectious disease or neoplastic disorder (Terrazzano et al., 2007).

### In vivo

Historically, monoclonal-mediated NK-ablation by anti-NK1.1 and anti-Asialo GM1 mAbs together with the use of murine models (i.e., SCID, NOD g γ-chain ko) have greatly contributed to the definition of NK functions. Nowadays, the elegant opportunity offered by the flox-cre inducible tissue specific genes regulation lead to even more tight observation of NK cells subsets associated receptors function in pathological settings (Eckelhart et al., 2011). However, NK cells regulation have been seldom analysed and manipulated in a global way. One possibility could be to reverse the ongoing experimental strategy: i.e., starting from reductionistic models gamma chain NOD ko mice where it is possible first to manipulate selectively NK cells compartment and then adding by cell transfer the other lymphocytes subsets.

## Conclusion

Despite the initial description of NK cells as lymphocytes of the innate response, recent progress has pointed out a broader role for these lymphocytes in immunity. In adaptive immunity, the NK polarize the activity of T lymphocytes, regulating also the adaptive response. In this regard, it is possible to speculate that the functions of NK cells have been developed not only to represent a “first line defense” and to generate the context of stimuli in innate response useful to foster the later stages of the specific response, but also to integrate, polarize, and complete the immune adaptive stages through an implementation of specific NK activities, such as the cytokine secretion, the interaction with the DC, the regulation of T-cell and B cells priming and activation, and, finally, the contribution to the “immunological memory.” These features make NK cells able to perform functions traditionally ascribed to the adaptive immunity. Therefore, NK cells seem to violate the classical paradigm that defines them just as innate immunity effectors cells. Currently, NK biology offers intriguing points of view to understand the complexity of the immune response architecture. In this sense, the blurring by NK cells of the frontier between innate and acquired immunity could provide new perspectives for the acquisition of more complete knowledge over the physiology of immune system and for the obtaining of immune-based therapeutical strategies.
